# Antiplatelet Therapy with Cangrelor in Patients Undergoing Surgery after Coronary Stent Implantation: A Real-World Bridging Protocol Experience

**DOI:** 10.1055/s-0040-1721504

**Published:** 2020-12-23

**Authors:** Roberta Rossini, Giulia Masiero, Claudia Fruttero, Enrico Passamonti, Elba Calvaruso, Moreno Cecconi, Cesare Carlucci, Marco Mojoli, Parodi Guido, Giuseppe Talanas, Simona Pierini, Paolo Canova, Nicoletta De Cesare, Stefania Luceri, Nicoletta Barzaghi, Giulio Melloni, Giorgio Baralis, Alessandro Locatelli, Giuseppe Musumeci, Dominick J. Angiolillo

**Affiliations:** 1 Ospedale Santa Croce e Carle, Cuneo, Italy; 2 Ospedale di Cremona, Struttura Complessa di Cardiologia, Cremona, Italy; 3 Ospedale di Civitanova Marche, Civitanova Marche, Italy; 4 Ospedale Santa Maria degli Angeli, Pordenone, Pordenone, Italy; 5 Azienda Ospedaliera Universitaria di Sassari, Struttura Complessa di Cardiologia Clinica ed Interventistica, Sassari, Italy; 6 P.O. BASSINI—ASST Nord Milano, U.O.C. Cardiologia, Milano, Italy; 7 ASST Papa Giovanni XXIII, Unità di Cardiologia 2, Bergamo, Italy; 8 Policlinico san Marco, IOB, Zingonia-Osio Sotto, Bergamo, Italy; 9 Cardiology Division, Ospedale Mauriziano, Torino, Italy; 10 University of Florida College of Medicine, Jacksonville, Florida, United States

**Keywords:** antiplatelet therapy, stent, surgery, bridging, cangrelor

## Abstract

**Objective** The aim of the study is to describe the real-world use of the P2Y _12_ inhibitor cangrelor as a bridging strategy in patients at high thrombotic risk after percutaneous coronary intervention (PCI) and referred to surgery requiring perioperative withdrawal of dual antiplatelet therapy (DAPT).

**Materials and Methods** We collected data from nine Italian centers on patients with previous PCI who were still on DAPT and undergoing nondeferrable surgery requiring DAPT discontinuation. A perioperative standardized bridging protocol with cangrelor was used.

**Results** Between December 2017 and April 2019, 24 patients (mean age 72 years; male 79%) were enrolled. All patients were at high thrombotic risk after PCI and required nondeferrable intermediate to high bleeding risk surgery requiring DAPT discontinuation (4.6 ± 1.7 days). Cangrelor infusion was started at a bridging dose (0.75 µg/kg/min) 3 days before planned surgery and was discontinued 6.6 ± 1.5 hours prior to surgical incision. In 55% of patients, cangrelor was resumed at 9 ± 6 hours following surgery for a mean of 39 ± 38 hours. One cardiac death was reported after 3 hours of cangrelor discontinuation prior to surgery. No ischemic outcomes occurred after surgery and up to 30-days follow-up. The mean hemoglobin drop was <2 g/dL; nine patients received blood transfusions consistent with the type of surgery, but no life-threatening or fatal bleeding occurred.

**Conclusion** Perioperative bridging therapy with cangrelor is a feasible approach for stented patients at high thrombotic risk and referred to surgery requiring DAPT discontinuation. Larger studies are warranted to support the safety of this strategy.

## Introduction

Dual antiplatelet therapy (DAPT) with aspirin and a P2Y _12_ inhibitor is the standard of care to prevent thrombotic complications in patients undergoing percutaneous coronary intervention (PCI) with stent implantation. 1 Perioperative management of DAPT in patients undergoing nondeferrable surgery still raises relevant safety concerns. On one hand, discontinuation of DAPT to reduce bleeding complications is associated with an enhanced risk of thrombotic events, while maintenance of DAPT to avoid perioperative thrombotic complications increases the risk of bleeding and the need for transfusions, which are both known determinants of poor prognosis. 2 A strategy of temporary transition with an intravenous antiplatelet agent may represent a desirable treatment option in patients deemed at high thrombotic risk undergoing nondeferrable relevant bleeding risk surgery requiring a predictable interruption of platelet inhibition. 3 4 5 6 The European Society of Cardiology (ESC) guidelines recommend a bridging strategy with an intravenous antiplatelet agent if both oral antiplatelet agents have to be discontinued perioperatively, especially within 1 month after PCI. 5 However, currently there are no antiplatelet agents approved by drug regulating agencies for such bridging indication.

Cangrelor is an intravenous, rapidly acting, and reversible potent P2Y _12_ platelet receptor antagonist approved for the reduction of thrombotic cardiovascular events in P2Y _12_ inhibitor naïve patients undergoing PCI. 7 8 Cangrelor also has a very short half-life (3–6 minutes) with an offset of its antiplatelet effects within 60 minutes making it an attractive option for bridging therapy. Accordingly, a dose-finding investigation was conducted to identify a regimen of cangrelor associated with a “thienopyridine-like” effect for bridging purposes, hence not as potent as that used for its PCI indication which is known to lead to near complete P2Y _12_ inhibition. Such identified bridging dosing regimen of cangrelor (0.75 μg/kg/min) was tested in a prospective randomized double-blind study among thienopyridine-treated patients undergoing coronary artery bypass grafting surgery (CABG) showing to achieve adequate levels of platelet inhibition during the washout phase from oral P2Y _12_ inhibitors until the time of surgery without safety (bleeding or nonbleeding) concerns. 9 However, there is limited reported data on the use of cangrelor among patients undergoing noncardiac surgery (NCS). 10 11 12 13 14 15 16 On this background, we report the results of a prospective, multicenter registry describing real-world experience of a prespecified bridging protocol using cangrelor conducted in patients referred to nondeferrable intermediate to high bleeding risk surgery requiring withdrawal of DAPT.

## Materials and Methods

### Study Population

We prospectively collected data from nine Italian centers that had included patients undergoing surgery using cangrelor as a bridging strategy in the perioperative phase ( Table 1 ). All patients were still on DAPT due to recent coronary stent implantation and required nondeferrable, intermediate to high bleeding risk surgery demanding discontinuation of one or both antiplatelet agents. Patients with prohibitive hemorrhagic risk profile or active bleeding were not considered suitable for bridging therapy with cangrelor. Guidelines indicate that the perioperative management of antithrombotic therapy needs to be defined in a multidisciplinary manner to better state the trade-off between ischemia and bleeding. 17 18 19 To predict the individual risk of thrombotic complications against the anticipated risk of surgical bleeding complications, a multidisciplinary consensus document among cardiologists, surgeons, and anesthesiologists on practical recommendations for standardizing management of antithrombotic therapy management in patients treated with coronary stents (Surgery After Stenting 2) was used ( https://itunes.apple.com/us/app/stent-surgery/id551350096?mt1⁄48). ^20,21^ Briefly, the document defined the thrombotic risk (low, intermediate, or high) by combining angiographic and clinical features, time from stent implantation, type of implanted stent, and perioperative need for DAPT discontinuation. Moreover, surgical bleeding risk was defined into low, intermediate, or high according to the inherent hemorrhagic risk of over 250 cardiac and noncardiac surgical procedures based on the amount of blood loss and the anticipated difficulty in achieving adequate local hemostasis. 20 21

**Table 1:** Baseline characteristics of the patient population and the management of cangrelor infusion during the perioperative phase

Patient	Age	No of stents	ACS	P2Yy _12_ inhibitor	Type of surgery	PCI-surgery time interval (days)	Reason for bridge	P2Y _12_ discontinuation time (days)	Surgery on aspirin	Discontinuation cangrelor-surgery time (hours)	Cangrelor reintroduction	Cangrelor reintroduction-surgery time (hours)	Bridge after surgery time (hours)
1	68	2	1	T	Lobectomy	76	A	5	1	9	1	24	12
2	79	2	1	T	Prostatectomy	80	A	5	1	7	1	18	12
3	63	2	0	C	CABG	7	B	5	1	10	0	–
4	74	5	1	C	Emicolectomy	21	B	6	1	6	1	6	108
5	60	2	0	C	Nasal surgery	257	C	6	0	8	1	12	24
6	76	3	1	T	Gastrectomy	51	A	5	1	6	1	6	72
7	83	4	1	T	MV replacement	6	B	2	1	8	1	8	41
8	54	1	T	Hip prostesys	658	D	6	1	9	1	7	24
9	52	1	P	CABG + valve repair	35	A	6	1	8	0	–
10	72	3	1	T	CABG + valve repair	13	B	5	1	8	0	–
11	81	1	T	CABG	51	A	6	1	8	1	8	61
12	73	6	1	T	AVR	210	D	5	1	8	0	–
13	71	1	T	Prostatectomy	40	A	5	1	6	1	8	66
14	79	3	1	C	Hip prostesys	48	A	5	1	8	1	9	12
15	69	3	1	T	Gastric endoscopy	39	A	5	1	3	0	–
16	69	1	T	CABG	2	A	2	1	3	0	–
17	66	1	T	CABG	2	A	2	1	3	0	–
18	59	1	T	CABG	2	A	2	1	3	0	–
19	87	1	C	ERCP	30	A	7	1	10	1	7	12
20	74	3	0	C	Nefrectomy	139	E	5	1	6	1	4	21
21	74	3	1	C	Gastrectomy	30	B	5	1	6	1	12	120
22	70	4	1	T	Limb amputation	57	A	4	1	3	0	–
23	81	3	0	C	Bladder endoscopic surgery	10	E	2	1	6	0	–
24	88	1	T	Renal endoscopic surgery	49	A	6	1	6	0	–

Abbreviations: A, ACS < 3 mo; ACS, acute coronary syndrome; AVR, aortic valve replacement; B, PCI < 1 mo; C, clopidogrel; C, stent failure occurrence; CABG, coronary artery by-pass graft; D, previous bioresorbable scaffold implantation; E, complex PCI; ERCP, endoscopic retrograde cholangiopancreatography; MV, mitral valve; P, prasugrel; PCI, percutaneous coronary intervention; ST, stent thrombosis; T, ticagrelor.

### Bridging Protocol

**Fig. 1:**
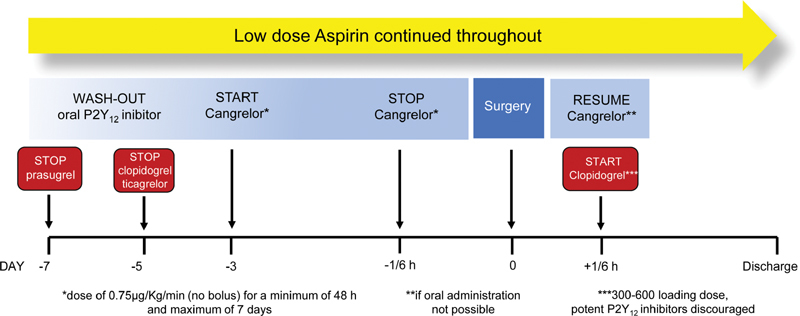
Standardized bridging protocol using cangrelor infusion before and eventually after nondeferrable surgery which required discontinuation of one or both antiplatelet agents in patients with previous PCI. 21 PCI, percutaneous coronary intervention.

A standardized bridging protocol using cangrelor infusion before and eventually after surgery was reserved for patients deemed at high thrombotic risk undergoing nondeferrable surgery at intermediate to high risk of bleeding, which requires a predictable interruption of platelet inhibition at the time of surgery 21 22 ( Fig. 1 ). According to International Guidelines, clopidogrel and ticagrelor were discontinued for 5 days before surgery, while prasugrel was discontinued for 7 days. 5 In line with expert consensus recommendation, cangrelor at a bridging dose regimen (0.75 μg/kg/min infusion without a bolus) was initiated 2 to 3 days following clopidogrel and ticagrelor discontinuation and 3 to 4 days after prasugrel discontinuation. 9 22 As a matter of fact, previous data reported a broad variability in platelet reactivity after discontinuation of thienopyridine therapy, meaning that a considerable number of patients are not adequately protected when stopping thienopyridine therapy for up to a week. 9 Cangrelor infusion was discontinued up 1 to 10 hours before surgery. Otherwise, according to BRIDGE Trial design, a lower P2Y _12_ inhibitor discontinuation time and PCI-to-surgery time intervals were allowed in patients referred to nonemergent CABG planned between 48 hours but no longer than 7 days following coronary stent implantation. 9 Since thrombotic complications occur most frequently soon after surgery, close clinical and electrocardiographic monitoring in an intensive care unit was emphasized in the postoperative period. Complete blood count assessments were performed daily to monitor hemoglobin levels. Once successful hemostasis was achieved, oral P2Y _12_ inhibiting therapy was resumed within 24 to 48 hours. Clopidogrel was preferred over prasugrel or ticagrelor in this setting of increased bleeding risk patients. In particular, to avoid a potential drug–drug interaction with cangrelor, clopidogrel 600 mg loading dose (LD) was administered immediately after discontinuation of cangrelor. 22 23 If oral P2Y _12_ inhibiting therapy was temporarily not administrable (i.e., in case of failed gastrointestinal function recovery or prolonged maintenance of drainages), intravenous infusion of cangrelor was resumed, after careful evaluation of the bleeding risk.

### Study End Points

Clinical events during the perioperative phase (up to 48 hours from surgery) and at 30-days follow-up were prospectively collected. Cardiac death, periprocedural and spontaneous myocardial infarction (MI), urgent target-lesion revascularization (TLR), and definite stent thrombosis (ST) were assessed according to the Academic Research Consortium Criteria and the American College of Cardiology/American Heart Association (ACC/AHA) cardiovascular end points data standards. 24 Major ischemic adverse cardiac events (MACE) were defined as the composite of cardiac death, MI, and TLR.

Bleeding events were defined using the Global Use of Strategies to Open Occluded Coronary Arteries (GUSTO) and Bleeding Academic Research Consortium (BARC) definition. 25 Major bleedings were defined as GUSTO-defined severe/life-threatening bleedings and the composite of BARC-defined type 3b, type 3c, and type 5 bleedings. For the purpose of the study, CABG-related bleeding (BARC type 4) was included in the major bleeding category in case of fatal bleeding, reoperation following closure of sternotomy for the purpose of controlling bleeding, transfusion of ≥5 units or relevant chest tube output. Mild bleedings were defined as GUSTO-defined moderate bleedings and BARC-defined type 3a. The amount of blood loss from drainages, the hemoglobin drops, and the need for blood transfusion were also assessed.

### Statistical Analysis

All efficacy and safety end points were collected during the perioperative phase (evaluated within 48 hours from surgery) and at 30 days follow-up. Quantitative variables were summarized as mean ± standard deviation, while categorical ones as count and percentages in each category.

## Results

### Patient Characteristics and Perioperative Management

**Fig. 2:**
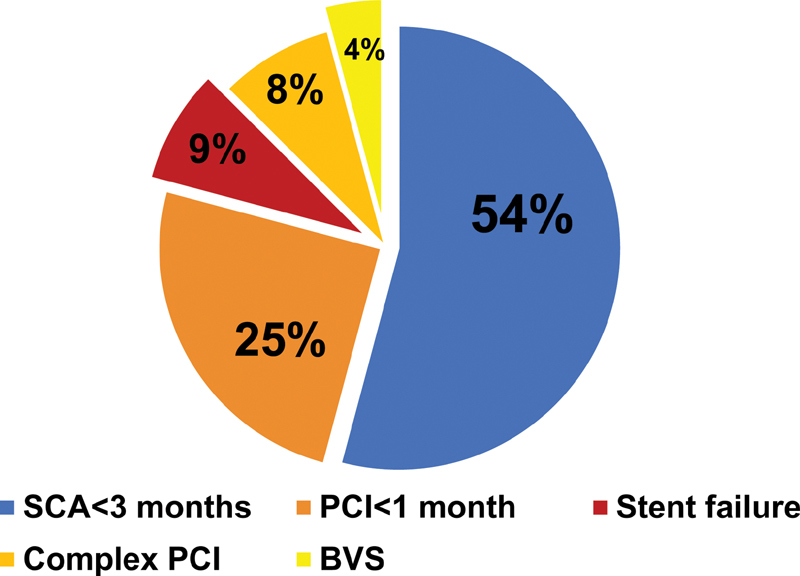
High thrombotic risk categories included in the studied patient population undergoing to bridging with cangrelor for nondeferrable relevant bleeding risk surgery.

**Fig. 3:**
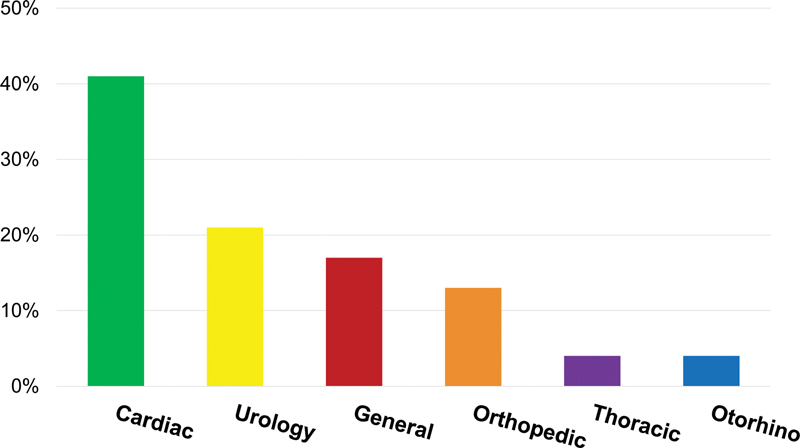
Nondeferrable, intermediate-high bleeding risk surgeries included in the studied patient population undergoing bridging with cangrelor for high ischemic risk.

Between December 2017 and April 2019, 24 patients were identified to be at high thrombotic risk and required nondeferrable intermediate-high bleeding risk surgery. Baseline characteristics of the patient population and data on cangrelor infusion during the perioperative phase are shown in Table 1 . Mean age was 72 ± 9 years and 79% were men. In the majority (83%) of patients, the index PCI was performed due to an acute coronary syndrome (ACS) and 2.1 ± 1.5 stents per patient were implanted. The average time from PCI to surgery was 80 ± 136 days. Two-thirds of the patients were on ticagrelor 90 mg bid ( *n* = 14, 58% of cases), one-third on clopidogrel ( *n* = 9, 38% of the cases), and a minority on prasugrel ( *n* = 1, 4% of cases). All patients were also on aspirin 100 mg/qd. According to a multidisciplinary evaluation and SAS2 criteria, 20 high thrombotic risk categories included: PCI within 1 month (patients number 3–4-7–10–21), ACS within 3 months (patients number 1–2-6–9-11–13 to 19–22–24), prior stent failure due to definite subacute ST (patient number 5), previous complex PCI with multiple stents implantation and left main involvement (patients number 20–23), and use of a bioresorbable vascular scaffold (BRS) (patients number 8–12) ( Fig. 2 ). Intermediate to high bleeding risk surgeries, deemed nondeferrable from a surgical point-of-view especially due to recently encountered neoplastic pathology, included: pulmonary lobectomy (patient number 1), prostatectomy (patients number 2–13), colectomy (patient number 4), endoscopic surgery (patients number 15–23–24), gastrectomy (patients number 6–21), nephrectomy (patient number 20), paranasal sinus surgery (patient number 5), hip replacement (patients number 8–14), endoscopy sphincterotomy (patient number 19), and limb amputation (patient number 22) ( Fig. 3 ). Eight patients (numbers 3–7-9 to 12–16 to 18) were planned for nonemergent CABG and valvular repair. The average time of P2Y _12_ inhibitor discontinuation was 4.5 ± 1.7 days prior to surgery. All patients but one maintained aspirin through the perioperative phase. Cangrelor infusion was started at the bridging dose (0.75 µg/kg/min) 2.9 ± 0.9 days before planned surgery and was discontinued 6.6 ± 1.5 hours prior to surgical incision. After surgery, drainages were left in all but six patients. In 55% of patients, cangrelor was resumed within 24 hours from surgery (mean time 8.6 ± 6.1 hours) for a mean of 39 ± 38 hours. Drainages were removed after discontinuation of cangrelor to reduce bleeding complications. Within 2 hours from postoperative cangrelor discontinuation, a 600 mg clopidogrel LD was administered in all patients. In all other patients, clopidogrel was resumed at 36 ± 22 hours after surgery once successful hemostasis was achieved.

### Outcomes

Adverse events are reported in Table 2 . Prior to surgery, one cardiac death occurred due to fatal ST elevation MI at 3 hours after cangrelor discontinuation. The patient was still on aspirin 100 mg/qd, whereas ticagrelor 90 mg/bid had been discontinued 5 days prior to nonemergent surgery. This 70-year-old female patient was afflicted by chronic limb-threatening ischemia (CLTI) with lower extremities rest pain (Radford stage IV). She experienced a failed attempt of revascularization complicated with an ACS treated with an effective complex PCI (four stents implanted) on the left main and left descending artery. Due to progression of CLTI disease to incapacitating pain and nonhealing wounds (Radford stage V) but with no signs of infection she was scheduled for a nonemergent but nondeferrable lower limb amputation 57 days after the PCI. 26 No other major ischemic adverse outcomes occurred up to 30 days follow-up. The average hemoglobin drop was 1.8 ± 1.8 g/dL and nine patients required periprocedural blood transfusions which was consistent with the type of surgery performed, known to need a larger spending of blood products, as orthopaedic, general, and cardiac surgery. 27 One-third of patients experienced periprocedural BARC 3/GUSTO moderate bleeding events due to drainages output, but a hemoglobin drop ≥3 g/dL was observed in only three cases. No fatal, life-threatening, or intracranial bleedings occurred. No patient required re-operation or experienced hemodynamic compromise due to bleeding.

**Table 2:** Clinical ischemic and bleeding events during the perioperative phase (up to 48 h from surgery) and at 30-d follow-up

	<48 h	>48 h to 30 d
Ischemic events		
MACE	1 (4%)	0 (0%)
Cardiac death	1 (4%)	0 (0%)
MI	1 (4%)	0 (0%)
Urgent TLR	0 (0%)
ST	1 (4%)	0 (0%)
Bleeding events		
GUSTO-defined severe/life-threatening	0 (%)
BARC-defined type 5	0 (%)
GUSTO-defined moderate	9 (38%)	4 (17%)
BARC-defined type 3a	9 (38%)	4 (17%)
BARC-defined type 3b-3c	3 (13%)	2 (8%)
Hemoglobin drop (g/dL)	1.8 ± 1.7	–
Need for blood transfusion	9 (38%)	4 (17%)

Abbreviations: BARC, Bleeding Academic Research Consortium; GUSTO, Global Use of Strategies to Open Occluded Coronary Arteries; MACE, major adverse ischemic events; MI, myocardial infarction; ST, stent thrombosis; TLR, target lesion revascularization.

Note: Variables are presented as count and percentages or mean values ± standard deviation.

## Discussion

To the best of our knowledge, this is the largest multicenter experience on a bridging protocol with cangrelor in stented patients at high ischemic risk undergoing nondeferrable intermediate-high bleeding risk surgery. In the present case series, a reassuring safety bleeding profile of cangrelor was demonstrated, with no identified fatal/life-threatening bleedings or major blood loss requiring re-operation occurring in the perioperative phase.

Compared with patients without CAD, those who have previous PCI are at higher risk for MACE when undergoing surgery. 6 28 29 The risk of an ischemic event (e.g., ST, MI, and cardiac death) associated with surgery is strictly dependent on time from PCI, patient's surgical and cardiac risk, and the need for DAPT interruption. 2 3 4 Moreover, surgery itself is associated with proinflammatory and prothrombotic effects regardless of previous stenting. 2 3 4 Several observational studies have reported that the surgical risk in PCI treated patients stabilizes after 3 to 6 months; furthermore, selected patients without high-risk clinical or lesion characteristics showed the same perioperative risk of patients without CAD already beyond the first month after any type of stent implantation. 21 30 In fact, current ESC guidelines recommend P2Y _12_ inhibitor discontinuation 1 month after PCI, irrespective of the stent type, if aspirin can be maintained throughout the perioperative period. 5 In patients with recent MI or other high ischemic risk features requiring DAPT, elective surgery should be postponed for up to 6 months. 5 A bridging strategy with an intravenous antiplatelet agent is required if both oral antiplatelet agents have to be discontinued perioperatively, especially within 1 month after PCI. 5 However, there are no specific recommendations on perioperative antiplatelet therapy provided in those patients with high ischemic risk features requiring nondeferrable high hemorrhagic risk surgery between 1 and 6 months from PCI. 5 18 19 As a matter of fact, maintaining antiplatelet therapy to minimize ischemic complications confers an increased risk of bleeding and need for transfusions, which are both determinants of poor prognosis, including higher mortality risk. 4 25 31 32 In this setting, a strategy of temporary transition with a potent and effective intravenous antiplatelet agent with a predictable and safe interruption of platelet inhibition may represent an appropriate treatment option. 4 5 6

Currently, the only intravenous antiplatelet agents available for clinical use and potentially usable for bridging include cangrelor and glycoprotein IIb/IIIa inhibitors (GPI). However, cangrelor is a more attractive agent for bridging. In fact, it has a very short half-life with rapid resumption of platelet function (within 60 minutes), it does not require renal dosing adjustments and has a specific dosing regimen for bridging identified from a dose-finding investigation aimed at achieving “thienopyridine-like” levels of platelet inhibition; moreover, cangrelor has been specifically tested in a prospective, randomized, double-blind, placebo-controlled trial of patients undergoing CABG. 9 33 Conversely, the short-acting GPIs (i.e., eptifibatide and tirofiban) have a longer half-life and slower offset of action (i.e., 4–6 hours) compared with cangrelor, require renal dosing adjustments, and are used at the ACS dosing regimen given that there is no dedicated bridging dose. 34 Moreover, GPIs are known to be associated with an increased risk of thrombocytopenia, particularly with prolonged infusion, a phenomenon associated with worse outcomes, including mortality. 35 Overall, these pharmacologic characteristics enhance the risk of bleeding complications associated with GPI use which has never been tested for bridging in a randomized study. 9 31 36 Accordingly, after a multidisciplinary assessment, we selected high thrombotic risk patients with previous PCI undergoing nondeferrable surgery. On one hand, these patients cannot safely interrupt oral antiplatelet therapy, on the other, they required a predictable interruption of platelet inhibition at the time of surgery to minimize blood loss. 5 21 We tailored this strategy mainly for patients with a recent PCI or ACS with a clear indication to urgent cardiac or NCS with intermediate to high bleeding risk. Subsequently, we applied a prespecified bridging protocol with cangrelor infusion with a strict patient monitoring during the perioperative phase. 22

To date, there is no randomized trial of bridging with cangrelor in NCS, and limited observational experience are available with heterogenous prescribing and monitoring practices, which may contribute to suboptimal outcomes. 10 11 12 13 14 15 16 Likewise, data on bridging use of short-acting GPIs are heterogeneous as well with regard to type of surgery, inclusion criteria, time windows between stent implantation or ACS and surgery, and antiplatelet strategies during the perioperative period which may partly explain the reported variability in success rates. 37 Despite the inclusion of a similar miscellaneous of surgical procedures, we believed that the strength of our study is a standardize case by case approach evaluation to weight the ischemic and bleeding risk and the use of a bridging protocol with a close postoperative monitoring which could have had a positive impact on our study findings. 19 20 Moreover, the possibility of restoration of a switch-on/switch-off infusion of a reversible antiplatelet agent (i.e., cangrelor) immediately after surgery when oral P2Y _12_ inhibiting therapy was temporarily not administrable, potentially allowed a safer monitoring in the postoperative phase when a careful balance between thrombotic and bleeding risk is particularly relevant. 20 As a matter of fact, differently from previously reported worrisome increase in bleeding rates during bridging therapy with cangrelor, 10 11 37 we showed no fatal/life-threatening bleedings or major blood loss requiring re-operation and a restrained mean hemoglobin drop. Additionally, one-third of patients experienced periprocedural BARC 3/GUSTO moderate bleeding events due to drainages output, mostly with a hemoglobin drop <3 g/dL and below the hemorrhages/transfusion rates (up 65%) reported in previous observational studies on GPIs. 37 We reported one cardiac death due to fatal ACS after cangrelor discontinuation 3 hours before surgery, which occurred after almost 2 months from PCI, while on aspirin. On one hand, these data underline the risk of DAPT discontinuation even beyond 1 month especially in patients treated with complex PCI. In these patients, bridging with cangrelor up to 1 hour prior to surgical incision should be considered, even though the risk of thrombotic events might occur in perioperative phase, when cangrelor in suspended. On the other, previous studies on bridging therapy with cangrelor and eptifibatide had already highlighted such high ischemic risk profile reporting up to 3 to 6% rates of death or MI. 37 To note, preoperative administration of tirofiban was associated with more favorable efficacy with the cost of increased bleeding complications, although different study designs and populations may partly or totally explain the variability in success rates reported in those studies. 37 Undoubtedly, a strict selection of patient eligible for a bridging strategy needs to be fulfilled, excluding cases with lower bleeding risk profile to avoid overestimation of bleeding complications occurrence in predictable intermediate-to-low bleeding risk surgeries. 20 38 Moreover, due to the well-known consistently elevated morbidity and mortality with no bridge therapy, 2 3 4 28 29 more studies are warranted to support the efficacy and safety of our proposed standardized bridging strategy by identifying the patient population that would receive the maximum clinical benefit. Two multicenter observational registries will assess the impact of DAPT discontinuation on ischemic and bleeding events in stented patients referred to nondeferrable surgery. The MARS registry (ClinicalTrials.gov Identifier: NCT03981835) will study the current perioperative DAPT management strategies in the United States, including bridging, and outcome data after NCS. The MONET Italian study (ClinicalTrials.gov Identifier: NCT03445273) will evaluate outcomes after any type of surgery according to the actual application of the SAS2 indications and the clinical and angiographic risk of previous PCI. Furthermore, the ongoing randomized, double-blind, placebo-controlled MONET BRIDGE study (ClinicalTrials.gov Identifier: NCT03862651) aims to assess the efficacy and safety profile of a bridging strategy with cangrelor in patients who discontinue DAPT before surgery within 12 months from coronary stent implantation.

## Limitations

Limitations of this study include the restricted number of patients and the heterogeneity between the enrolled patients and type of surgeries. However, prescribing and monitoring practices were standardized leading to more homogenous and consistent patient management which could have contributed to our overall favorable observations. Nevertheless, albeit within a certain time window, exact timing of initiation and discontinuation of cangrelor infusion was left at the discretion of the treating physician. Furthermore, any perioperative platelet function test and transfusion was suggested. Despite the lack of evidence to guide the prophylactic use of platelet transfusions before major surgery, they should be considered according to the threshold of platelet count and in case of severe thrombocytopenia or critical bleeding. 39 40 Moreover, due to a large individual variation in the magnitude and duration of the antiplatelet effect, platelet function testing may be considered to help guide timing of starting cangrelor infusion after a P2Y _12_ discontinuation, especially in case of planned cardiac surgery. 5 22 Finally, data interpretation warrants caution due to the lack of a control group. Despite the abovementioned limitations, we think that our paper may add useful information to the current literature, reporting for the first time a strategy with a standardize case by case approach evaluation to weight the ischemic and bleeding risk and the use of a bridging protocol with a close postoperative monitoring. Moreover, this data might help to identify the patient population that would receive maximum benefit from bridging antiplatelet therapy, determine optimal administration strategy, monitoring therapy, and management of adverse events to delineate large prospective studies which are required in such challenging setting. 41

## Conclusion

Perioperative antiplatelet bridging therapy with cangrelor is a feasible approach for stented patients at high thrombotic risk referred to nondeferrable intermediate-high bleeding risk surgery requiring DAPT discontinuation. Larger studies are warranted to support the safety of this strategy.
